# Latent Autoimmune Diabetes in an Adult Male Presenting With Diabetic Ketoacidosis (DKA)

**DOI:** 10.7759/cureus.33847

**Published:** 2023-01-16

**Authors:** Syed K Imam, Fatima M Hassan, Hossam Mohamed

**Affiliations:** 1 Internal Medicine Department, Sultan Bin Abdulaziz Humanitarian City, Riyadh, SAU; 2 Nursing Department, Sultan Bin Abdulaziz Humanitarian City, Riyadh, SAU; 3 Intensive Care Unit, Sultan Bin Abdulaziz Humanitarian City, Riyadh, SAU

**Keywords:** latent autoimmune diabetes in adults, diabetic ketoacidosis, glutamic acid decarboxylase antibodies, type 1 diabetes, type 2 diabetes

## Abstract

Latent autoimmune diabetes in adults (LADA) is a common but not well-studied entity and its features overlap between type 1 and type 2 diabetes mellitus (T1D, T2D). Although autoimmunity is a well-known factor associated with this diabetes subtype, environmental factors including excessive weight, physical inactivity, and smoking may also be associated with it. It is commonly misdiagnosed as T2D and generally treated by oral anti-diabetes medications that cause a delay in commencing insulin therapy. There are few cases mentioned in the literature of LADA presenting first time as diabetic ketoacidosis (DKA). Here, we report a case of latent autoimmune diabetes in an adult male who presented with DKA.

## Introduction

Latent autoimmune diabetes in adults (LADA) displays similar autoantibodies like type 1 diabetes mellitus (T1D) but, in fact, is a milder and slowly progressive disease as compared to T1D. Therefore, LADA patients generally do not require insulin for some time following diagnosis [1,2]. As compared to type 2 diabetes mellitus (T2D), LADA patients demonstrate less insulin secretion and progress rapidly to insulin dependency [3]. Data from the United Kingdom Prospective Diabetes Study (UKPDS) showed that among patients who were diagnosed with T2D, 84% of those who were glutamic acid decarboxylase (GAD) antibody-positive at diagnosis progressed to insulin-requiring diabetes within six years compared to 14% of GAD antibody-negative individuals with T2D [3]. Autoimmunity seems to be the first pathological factor in LADA. Although T1D is characterized by a cluster of islet cell autoantibodies, LADA patients are mainly characterized by positive anti-GAD antibodies [4,5]. Besides autoimmunity, some other factors may also play roles in LADA and insulin resistance might contribute to some extent to the pathogenesis of LADA [6,7]. Patients with LADA generally do not manifest with diabetic ketoacidosis (DKA) at presentation owing to the slow progression of β-cell dysfunction. Here, we report a case of latent autoimmune diabetes in an adult male who presented with DKA.

## Case presentation

A 37-year-old Egyptian male, who was a smoker, came to the emergency room with recurrent vomiting for four hours, sore throat for two days, polyuria and polydipsia for one month, which became worse in the last two days, and four kg weight loss in two months. His father was diagnosed with T2D at the age of 60 years. He was born to a non-consanguineous parent, had four children, and all were free of disease. He had no history of using steroids or any other medication.

At the time of the presentation, he was conscious and well-communicative. His weight was 74 kg, height 171 cm and BMI was 25.31 kg/m^2^. Vital signs revealed blood pressure 128/87 mmHg, pulse 68/min, temperature 37.2°C, respiratory rate 19/min, and oxygen saturation 96% in room air. He had no evidence of acanthosis nigricans and the rest of the physical examination was unremarkable except for the pharynx, which appeared to be mildly inflamed. The abdomen was soft and non-tender. Gut sounds were audible and there was no organomegaly. Chest, precordial examination, and central nervous system assessment remained unremarkable. Relevant investigations were ordered and the results were as follows: white blood count (WBC) 7.15 10^3^/uL (normal 4.5-11 10^3^/uL), hemoglobin 15.3 g/dl (normal 13.5-17.5 g/dl), serum creatinine 60.4 µmol/l (normal 71-133 µmol/l), serum blood urea nitrogen 3.1 mmol/l (normal 3.2-7.1 mmol/l ), serum sodium 145.5 mmol/l (normal 137-145 mmol/l), serum potassium 4.0 mmol/l (normal 3.5-5.1 mmol/l), serum chloride 102 mmol/l (normal 98-107 mmol/l), serum bicarbonate 9.4 mmol/l (normal 22-26 mmol/l), random serum glucose 397 mg/dl (normal < 140 mg/dl), pH 7.23 (normal 7.35-7.45), partial pressure of CO_2_ (PCO_2_) 22.2 mmHg (normal 35-45 mmHg), partial pressure of O_2_ (PaO_2_) 88 mmHg (normal 80-100 mmHg), oxygen saturation 95% in room air (normal 97-100 mmHg), urine ketones 3+ (normal=negative), anion gap 38 mmol/l (normal < 12 mmol/l), Hemoglobin A1c (HbA1c) 14% (normal 4-5.7%), and TSH 1.23 miu/l (normal 0.35-4.9 mIU/l). Chest X-ray and ECG findings were normal.

Based on clinical and biochemical features, he was treated as a case of DKA and received a normal saline 1,000 ml infusion in one hour followed by 500 ml in the next hour, and intravenous normal saline infusion was continued at the rate of 250 ml/hour for four hours then 125 ml/hour infusion for next 24 hours. Potassium chloride 20-40 mEq was also added to saline infusion to keep serum potassium level within normal range. Human regular insulin was initiated at the rate of 0.1 units/kg/hour infusion and the dose of insulin was adjusted according to blood glucose level. Low-molecular-weight heparin 40 mg subcutaneously once daily was given to prevent the thromboembolic event. He responded well to medical management and the anion gap returned to normal, pH normalized, and glucose level came to normal range within 48 hours. During the ICU stay, he became free of vomiting and started eating normally. At this stage, intravenous insulin infusion was stopped and changed to subcutaneous basal insulin glargine 25 units at bedtime and bolus insulin aspart five units three times daily with correction doses every eight hours according to a subcutaneous sliding scale.

Because of a late onset, history of smoking and overweight status, LADA was strongly suspected and anti-GAD antibodies, anti-islet cell antibodies, and C-peptide levels were requested. The results reported as follows: anti-GAD 30 IU/ml (normal < 17 IU/ml), negative islet cell antibodies (normal=negative), and C-peptide 0.60 ng/ml (normal 1.1-4.4 ng/ml). Based on clinical, biochemical, and immunological features, he was diagnosed as LADA presenting first time with DKA. During the hospital stay, he received diabetes education, dietary advice, and psychological counseling and was discharged on insulin glargine 18 units at bedtime, and insulin aspart four units three times daily with a follow-up appointment in the endocrine clinic after four weeks. During outpatient follow-up, analog basal and bolus insulin were changed to biphasic human insulin 20 units at breakfast and 10 units at dinner for the sake of convenience and cost issues.

During the visit to his native country and follow-up in a clinic, his primary care physician recommended stopping insulin and initiating oral anti-diabetes medications. Within three days of initiating oral anti-diabetes medications, he developed severe hyperglycemia. However, he achieved reasonable glycemic control when insulin was reinstituted.

His current insulin regimen was comprised of biphasic human insulin, 25 units at breakfast and 20 units at dinner, and he reported having good glycemic control.

## Discussion

LADA is a common but not well-studied entity and its features overlap between T1D and T2D. The term "LADA" was first introduced in 1993, and it was discovered that this subgroup of patients shared phenotypical features with T2D and immunological features with T1D [8]. LADA accounts for 3-12% of all cases of diabetes in adults and is more frequent in Europe than in other parts of the world [3,5,8].

Although patients with LADA generally do not require insulin at the beginning of diagnosis, within six years of onset of the disease, β cells become severely impaired and result in insulin dependency in most LADA patients. Complete β-cell failure may take up to 12 years in such cases [9-10].

According to the Immunology of Diabetes Society, there are three main criteria to diagnose LADA: (1) adult age of onset (>30 years); (2) presence of any islet cell autoantibody; and (3) absence of insulin requirement for at least six months after diag­nosis [11]. However, the definition of LADA remains controversial and an open debate regarding these diagnostic criteria still exists.

Table 1 shown below depicts the clinical and biochemical findings of reported cases of LADA with DKA as compared to our case. All the reported cases in Table 1 were related to women, while our case was related to a man, which was also characterized by a positive family history of diabetes and early age of presentation as compared to other cases. None of the cases had a prior history of diabetes mellitus and all cases presented with DKA with poor glycemic control as evidenced by higher HbA1c.

**Table 1:** Clinical and biochemical findings to reported cases of LADA with DKA as compared to our case GAD: Glutamic acid decarboxylase; ICA: Islet cell antibodies; IAA: Insulin autoantibodies; NA: Not available; UTI: Urinary tract infection; DKA: Diabetic ketoacidosis; WBC: White blood count; ABG: Arterial blood gas; LADA: Latent autoimmune diabetes in adults; PCO_2_: partial pressure of CO_2_

	Case 1	Case 2	Case 3	Our case
	Ray S et al. [12]	Nadhem O, Nakhla E, Smalligan RD [13]	Gutch M et al. [14]	
Age	66	54	40	37
Gender	Female	Female	Female	Male
Presentation	Disorientation and confusion	Abdominal pain, nausea, vomiting, diarrhea	Abdominal pain, nausea, vomiting, drowsiness	Recurrent vomiting, sore throat, weight loss
Associated diseases	Impaired glucose tolerance	Hypothyroidism, bipolar disorder	Hypothyroidism	None
Family history of diabetes	No	No	No	Positive
Physical finding	Fever, tachycardia, tachypnea	Abdominal tenderness	Tachypnea, Kussmaul’s respiration	Pharyngeal congestion
BMI (normal 18-25 kg/m^2^)	19	35	28	25.3
Anion gap (normal <12 mmol/l)	High	21	25.8	38
Random serum glucose (normal < 140 mg/dL)	609	337	540	397
HbA1c (normal 4-5.7%)	9.5	13.7	10.3	14
Urine ketones (normal=negative)	Present	Present	Present	Present
ABGs				
pH (normal 7.35-7.45)	Acidosis	7.25	7.23	7.23
HCO3 (normal 22-26 mmol/l)	NA	12	NA	9.4
PCO_2_ (normal 35-45 mmHg)	NA	27.9	27	22.2
WBC (normal 4.5-11 10^3^/uL)	NA	14.8	12,3	7.15
C-peptide (normal 1.1-4.4 ng/ml)	0.19	0.34	0.2	0.6
Diabetes autoimmune markers				
Anti-GAD (normal <17 IU/mL)	159.18	> 30	>50	>30
ICA (normal=negative)	NA	NA	NA	Negative
IAA (normal=negative)	NA	Negative	NA	NA
Precipitating factors	UTI	Gastroenteritis	None	Pharyngitis
DKA management	Insulin and IV fluid	Insulin and IV fluid	Insulin and IV fluid	Insulin and IV fluid
Discharge with insulin	Yes	Yes	Yes	Yes

Hypothyroidism was found in two reported cases and one case is found to have impaired glucose tolerance, however, our case had no associated disease. Infection was found as one of the commonest precipitating factors and was seen in all cases except one in which no precipitating factor was identified. Furthermore, our case had no associated diseases as compared to other reported cases. T1D is characterized by the presence of multiple autoantibodies. However, anti-GAD is the commonest autoimmune marker present in LADA and found positive in all reported cases shown in Table 1. All the cases were managed with intravenous insulin and saline infusion and discharged home on subcutaneous insulin therapy.

Family history of T1D and T2D increases the risk of LADA by six-fold and two-fold respectively [15]. Genetically triggered autoimmunity is the first step towards LADA development that results in the slow destruction of β cells of the pancreas and reduction in insulin synthesis and release. Environmental factors such as smoking, being overweight, and obesity will lead to increased insulin resistance and a compensatory increase in insulin production that may ultimately lead to β-cell exhaustion and failure, resulting in hyperglycemia and LADA manifestation. LADA is genetically related to both T1D and T2D, but the strongest genetic risk locus is shared with T1DM. Researchers have observed a new independent gene (PFKFB3) at the known T1D locus which encodes glycolysis and insulin signaling in T2D and found that the PFKFB3 gene was closest to LADA and was most likely to be a functional gene candidate [16]. Genetic markers such as HLA-DR3 and DR4 antigens are commonly associated with LADA but HLA-DR4-DQ8 antigen is seen less commonly in LADA as compared to T1D, which causes more rapid β-cell destruction and insulin deficiency. Therefore, patients with LADA do not require insulin as readily as those with T1D, at least within the first few months of diagnosis and are less susceptible to developing acute complications during this time as compared to T1D.

So far, there is no specific guideline available for the treatment of LADA and these patients are mostly treated as a T2D, especially those who possess phenotype and biochemical features similar to T1D than T2D [17]. Furthermore, consistent data from randomized clinical trials highlight the importance of early initiation of insulin therapy in LADA regardless of the presence of some endog­enous insulin secretion [18]. Some studies have shown that insulin treatment, as well as dipeptidyl peptidase 4 (DPP-4) inhibitors, can sustain residual β-cell function, whereas sulphonylurea may accelerate β-cell failure and should not be used as first-line therapy in patients with LADA [19].

## Conclusions

LADA is a subtype of diabetes characterized by genetic, phenotypic, and immunological heterogeneity. It is important to catch the symptoms at the earliest stage because diagnosis of LADA at a later stage increases the risk of developing diabetes complications such as DKA. Therefore, diagnosing LADA in early stage is very crucial and a careful history including personal and family history of autoimmunity and diabetes must be taken and autoimmune LADA markers should be recommended in suspected cases. LADA shows variable rate of β-cell destruction and different de­grees of insulin resistance and autoimmunity. Therefore, an intervention intended to preserve β-cell function and early insulinization should be pursued in patients with LADA and personalized therapy should be implemented.
